# Collar Button Abscess

**Published:** 2016-02-11

**Authors:** Elle Kalbfell, Nicholas S. Adams, William T. Cullen

**Affiliations:** ^a^Michigan State University College of Human Medicine, Grand Rapids, Mich; ^b^Grand Rapids Medical Education Partners Plastic and Reconstructive Surgery Residency, Grand Rapids, Mich; ^c^Hand Surgery Centre, Grand Rapids, Mich

**Keywords:** collar button abscess, infections of the hand, web space, dorsal volar infection, traumatic hand injury

## DESCRIPTION

**Figure 1:**
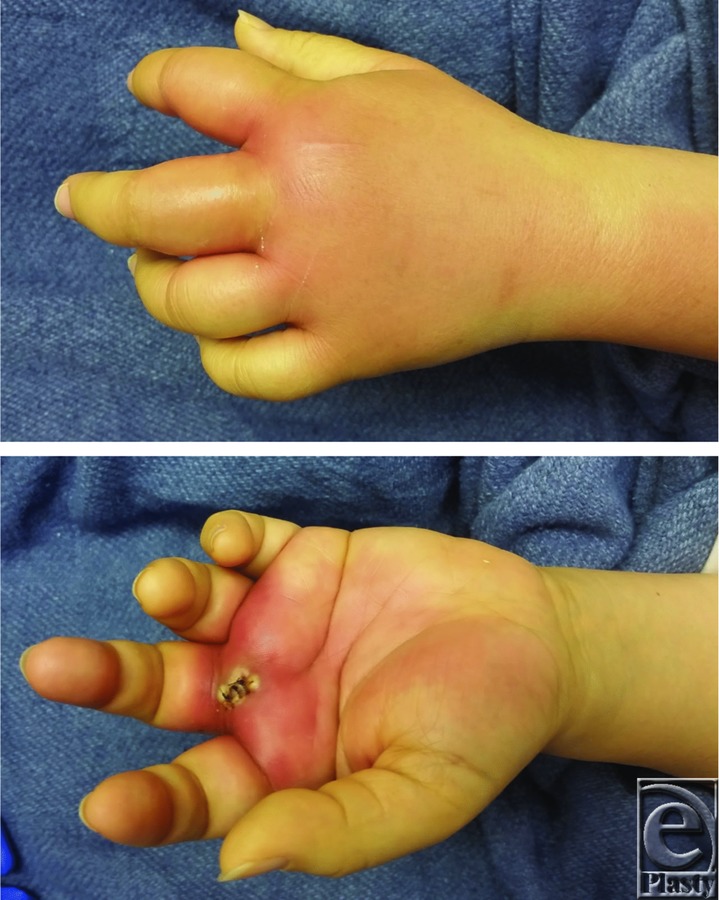
Puncture wound overlying the volar base of the long finger. The formation of a collar button abscess can be seen on both the dorsal and volar sides of the hand. The long and index fingers are abducted at rest.

A 52-year-old woman presented to the emergency department with 2 days of worsening left-hand pain after sustaining a puncture wound while cutting vegetables. The left hand was erythematous and swollen. The index and middle digits were in an abducted position at rest (Fig 1). The patient was started on intravenous antibiotics and underwent incision and drainage.

## QUESTIONS

1. **What are collar button abscesses?**
2. **What are the deep spaces of the hand?**
3. **What basic principles should be followed when treating collar button abscesses?**
4. **What potential causes and complications exist with deep infections of the hand?**

## DISCUSSION

A collar button abscess is a type of web space infection that involves the palmar and dorsal hand (Fig 1). It typically arises from a fissure, callus, or penetrating injury to the volar aspect of the hand.^1^ The strength and rigidity of the palmar aponeurosis prevent further volar extension but promote dorsal encroachment. The infection forms a communicative tract from the palmar fascia over the superficial transverse metacarpal ligament between the metacarpals to involve the dorsal subcutaneous space. As the infection travels dorsally through a fascial hole, the involved web space becomes swollen and inflamed, which results in abduction of adjacent digits and the characteristic “V” configuration with the apex pointing to the site of infection (Fig 1).^1^^-^3 Purulence within the dorsal subcutaneous space alone will not result in finger abduction. Patients presenting with a collar button abscess complain of pain and tenderness in the distal palm and web space.^1^ The natural palmar concavity may be lost.^2^ Collar button abscesses are aptly named, as they resemble the collar buttons used to fasten the collars of men's dress shirts in the early 20th century.^1^

**Figure 2:**
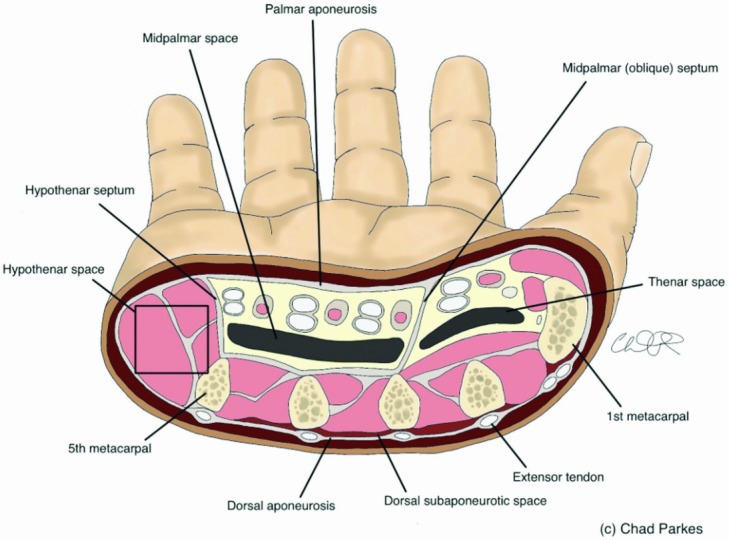
Cross-sectional anatomy demonstrating the deep spaces of the hand.

Common hand infections can be divided into superficial and deep with regard to the fascial planes and compartments of the hand. It was not until the mid-1900s, under the study of Dr Allen Kanavel, that the compartments within the hand were well understood. Today, deep-space infections comprise 5% to 15% of all hand infections.^1^ These spaces include the interdigital web space and dorsal subaponeurotic, hypothenar, midpalmar, and thenar spaces (Fig 2). The interdigital web space comprises the palmar aponeurosis and overlying fat/skin. The space connects to the dorsal hand and is confined by the metacarpophalangeal joint and extensor tendons. The dorsal subaponeurotic space is located deep to the dorsal aponeurosis and superficial to the interosseous muscles. Containing the hypothenar muscles, the hypothenar space is unique in that it does not contain any flexor tendons. The borders of the midpalmar space include the flexor tendons of the ulnar 3 digits along with the midpalmar septum, hypothenar septum, and interosseous muscles. An abscess in this space will be represented by pain with movement of the middle and ring fingers. Finally, the thenar space lies beside the lateral fibrous septum, superficial to the adductor pollicis, and is separated from the midpalmar space by the midpalmar septum. All deep-space infections are surgical emergencies and require urgent irrigation and drainage. Typically, both volar and dorsal incisions are used over the affected compartment and care is taken to avoid crossing the apex of the web space.

**Figure 3:**
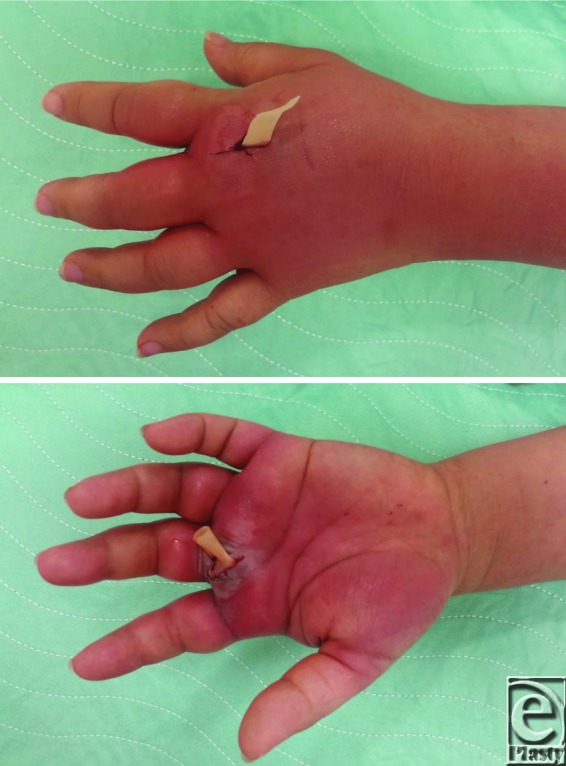
Postoperative photographs following incision and drainage through a dorsal and volar approach. A Penrose drain was left in place to allow for continued drainage of the abscess.

Surgical technique is of great importance when treating a collar button abscess for which surgery is always indicated. The collar button abscess always requires both volar and dorsal incisions due to the communicating channel between the volar and dorsal hand, which creates an hourglass-shaped abscess.^1^ Many incision patters exist, but treatment requires 2 incisions, a longitudinal incision between digits on the dorsal hand and a volar “Z-pattern” incision.^2^ These incisions should extend between the proximal edge of the web and distal palmar crease to avoid the common digital neurovascular bundle. Careful avoidance of a transverse web space incision due to the risk of scar contracture and loss of digital abduction is very important.^1^^,^^3^ Regardless of the incision type, blunt dissection should be used to create a connection between the dorsal and volar extensions of the abscess to avoid damaging the surrounding neurovascular bundles. After the collection of cultures and extensive irrigation, the incisions can be left open with the assistance of moist packing or a Penrose drain (Fig 3).^1^^,^^2^ Drains are typically removed after 24 to 48 hours and followed by hand soaks. Antibiotic therapy serves as an adjunct to surgical intervention.^3^

Collar button abscesses occur from direct inoculation or secondary infection from adjacent anatomical structures.^1^^-^3 *Staphylococcus aureus* and group A *Streptococcus* are the most common infectious agents, with *S aureus* reported to be 30% to 80% of positive cultures.^4^ Closed-space hand infections such as a collar button abscesses should be drained with urgency. Delay in treatment could result in the spread of infection throughout the deep spaces, risking significant compromise of hand function. Damage to neurovascular structures is possible with incision and drainage, and care should be taken when draining a collar button abscess due to the proximity of the bifurcating common digital nerves and arteries. As stated earlier, web space incision should never span transversely across the web space due to scar contracture and subsequent loss of finger abduction.^2^

Deep-space infections, including collar button abscesses, comprise a relatively small percentage of hand infections, but their clinical significance remains high. This article describes many of the specific technical pearls that are essential for the adequate treatment of collar button abscesses.
